# Occurrence of Different Gynandromorphs and Ergatandromorphs in Laboratory Colonies of the Urban Ant, *Monomorium floricola*

**DOI:** 10.1673/031.011.0117

**Published:** 2011-02-14

**Authors:** Ana E. de C. Campos, Luciane M. Kato, Maria F. M. de Zarzuela

**Affiliations:** ^1^ Instituto Biológico, Unidade Laboratorial de Referência em Pragas Urbanas. Av. Conselheiro Rodrigues Alves, 1252, Vila Mariana, São Paulo, SP. CEP: 04014-002; ^2^ Universidade Estadual Paulista — Júlio de Mesquita Filho, Instituto de Biociências — Campus de Rio Claro. Av. 24A, n. 1515, Bela Vista, Rio Claro, SP. CEP: 13506–900

**Keywords:** Formicidae, longevity

## Abstract

Colonies of *Monomorium floricola* (Jerdon) in laboratory conditions showed gynandromorphic and ergatandromorphic specimens, the former with nine different combinations of male and female tissues and the latter with 6 different combinations. Their development from egg to adult was around 74.6 days for gynandromorphs, and 87.5 days for ergantandromorphs.

## Introduction

Gynandromorphs are organisms that carry both male and female characteristics (Wheeler 1919; Donisthorpe 1929). A gynandromorph can have bilateral symmetry, one side female and one side male. Or mosaic, in which case the two sexes are not defined as clearly.

Among ants many combinations of male and female can occur: queen-male (gynandromorph), worker-male (ergatandromorph) and soldier-male (dynergatandromorph). Combinations of worker, queen and male are also known (Donisthorpe 1929). Such intercastes have been classified as gynergatandromorphs, ergatogynandromorphs, androgynergatomorphs, and androergatogynomorphs (Berndt and Kremer 1983).

Donisthorpe (1929) described 49 gynandromorphism cases in ants, including two reports on *Monomorium floricola* (Jerdon) (Myrmicinae). Scupola (1994) reported that until 1986 the number of reported gynandromorphisms increased to 86 and after that, other reports were published. Examples of gynadromorphisms are found in *Lasius (Acanthomyops) latipes* (Walsh) and *Camponotus* (*Colobopsis*) *albocinctus* (Ashmead) (Wheeler 1919), *Solenopsis invicta* (Buren) (Hung et al. 1975), *Solenopsis aurea* (Wheeler) (Cokendolpher and Francke 1983), *Monomorium pharaonis* (Linnaeus) (Berndt and Kremer 1983; Berndt and Eichler 1987), *Pheidole dentata* (Mayr) (Jones and Phillips-Jr 1985), *Pogonomyrmex occidentalis* (Cresson) (Taber and Francke 1986), *Smithistruma* (Munsee 1994), and *Myrmica sabuleti* (Meinert) (Scupola 1994).

Gynandromorphism in insects has been genetically and histologically studied and some hypotheses try to explain the phenomenon including anomalies in fertilization and cytogenesis irregularities during the embryogenesis (Jones and PhillipsJr 1985). According to Donisthorpe (1929), the possible cause of gynandromorphism in ants may be a result of two eggs that have joined to form a single egg with two nuclei. Such eggs may have originally been formed from different sexes, or they may have become different neither through the fertilization of one nor the fertilization of the other. Another possible cause is that the nucleus of a single egg can prematurely suffer cleavage and receive the spermatozoid later. The cleaved nucleus may be joined with a different fecundated nucleus or can, in part, parthenogenetically develop itself and in part suffer fertilization. It can still be possible that the gynandromorph's characteristics can result from trophic disturbance during the postembryonic development.

Ants have a haplodiploid sex determination and hypotheses that favor the explanation of the gynandromorphism are the effect in the dosage of the genes (homozygosis in some loci) and chromosomic not-disjunction during the beginning of embryogenesis (Taber and Francke 1986). According to Munsee (1994) gynandromorphism in insects occurs when some cleaved nuclei are males and others are females. Such nuclei, when reaching the cortical zone of the egg, become definitive to a certain part of the body and parts of one sex or the other are developed depending on the constitution of the cells that to be formed. Thus, entire regions or structures can show characteristics of one sex or another.

*M. floricola* is considered one of the most prevalent ant species in urban areas in Brazil (Bueno and Campos-Farinha 1999), mainly in hospitals, where they can act as vectors of pathogenic microorganisms (Fowler et al. 1993; Bueno and Fowler 1994). This species comes from Asia, it is polygynic, with monomorphic workers (Passera 1994) and apterous virgin queens (Smith 1965), (i.e. ergatogyne). Since a large number of gynandromorphs and ergatandromorphs were observed in our artificial nests, this paper contributes to the description of the diversity of external morphology found in such organisms.

## Materials and Methods

*M. floricola* were reared in artificial nests of different sizes made with microscopic slides and test tubes covered with red cellophane and fed with honey, water, and *Tenebrio molitor* (Linnaeus) (Coleoptera: Tenebrionidae) larvae were maintained in the laboratory under environmental conditions during the seasons of the southern hemisphere: autumn/winter (April to July) and spring/summer (September to December) 2002. This experiment was begun to evaluate the development of *M. floricola*, but when gynandromorphic and ergatandromorphic specimens were unexpectedly observed they were incorporated into a larger study.

To better illustrate and compare the several specimens found, a camera lucida joined to a stereomicroscope (Leica, Wild M10, www.leica-microsystems.com) was used.

## Results

In the autumn/winter evaluation of *M. floricola* development, five of the seven observed colonies that produced reproductive individuals (female ergatogynes and males) also produced gynandromorphic specimens and two colonies produced ergatandromorphic ones. In one colony, the number of gynandromorphs (n=5) was larger than the number of males (n=2) and ergatogynes (n=2).

In this species only ergatogynes were found and alate queens were never observed. A11 males were alate.

Gynandromorphs showed a mean development from egg to adult of 74.6 days, whereas ergatandromorphic specimens developed in 87.5 days. In the seasons of spring/summer, only two gynandromorphs developed in two different colonies with a mean development from egg to adult of 65 days. Observations of other artificial nests maintained at the Entomology Laboratory of Instituto Biológico, São Paulo, and at the Center of Studies of Social Insects (CEIS/UNESP) in Rio Claro, also showed a great number of gynandromorphic and ergatandromorphic specimens, ranging from one to 58 individuals found weekly in the polidomic nests.

**Figure 1.**
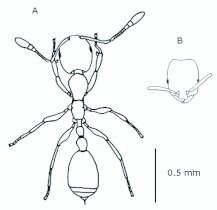
*Monomorium floricola* worker. (A) Dorsal view, (B) head frontal view. High quality figures are available online.

**Figure 2.**
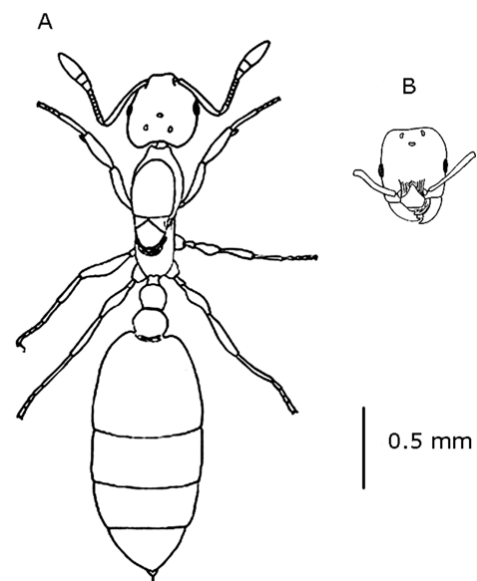
*Monomorium floricola* ergatogyne. (A) Dorsal view, (B) head frontal view. High quality figures are available online.

**Figure 3.**
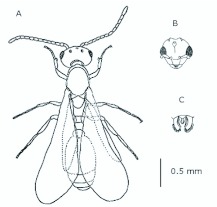
*Monomorium floricola* male. (A) Dorsal view, (B) head frontal view, (C) genitalia — frontal view. High quality figures are available online.

*M. floricola* workers were apterous, were 2mm long and had geniculate antennae with 12 segments and the ocelli were absent (Figure 1). Ergatogynes were apterous since their emergence (Wheeler 1919) and had 12-segment geniculate antennae, the eyes were small when compared to male eyes, there were three ocelli and the alitrunk was light brown (Figure 2). Males were alate with 13-segment antennae with reduced scape, eyes and ocelli were larger than those in the ergatogynes and the alitrunk was dark brown in color (Figure 3).

**Figure 4.**
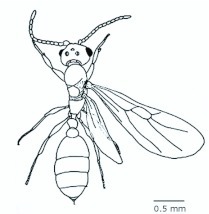
*Monomorium floricola* gynandromorph with ergatogyne characteristics on the left side of alitrunk and gaster. High quality figures are available online.

**Figure 5.**
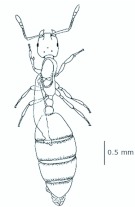
*Monomorium floricola* gynandromorph with ergatogyne gyne characteristics on the head and right side of alitrunk. High quality figures are available online.

**Figure 6.**
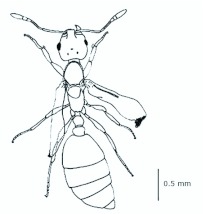
*Monomorium floricola* gynandromorph with ergatogyne characteristics on the head and gaster except by the left eye. High quality figures are available online.

**Figure 7.**
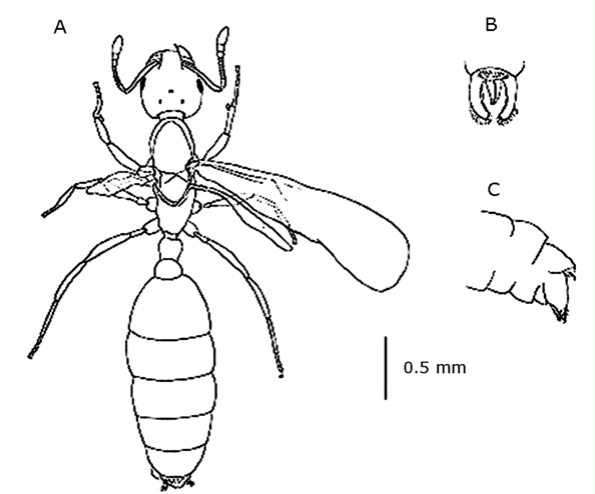
*Monomorium floricola* gynandromorph with ergatogyne characteristics on the head and left side of alitrunk except by vestigial wing. (A) Dorsal view, (B) genitalia, (C) genitalia lateral view. High quality figures are available online.

**Figure 8.**
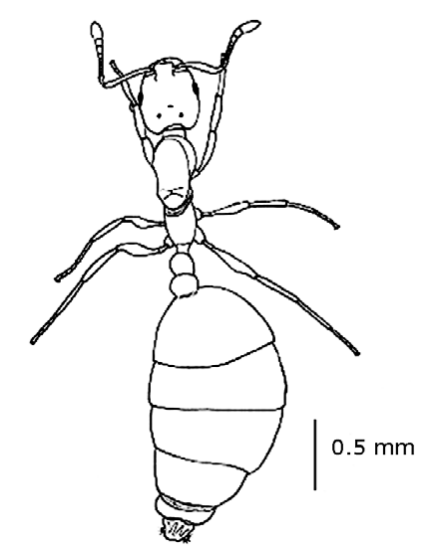
*Monomorium floricola* gynandromorph with predominance of ergatogyne characteristics. High quality figures are available online.

**Figure 9.**
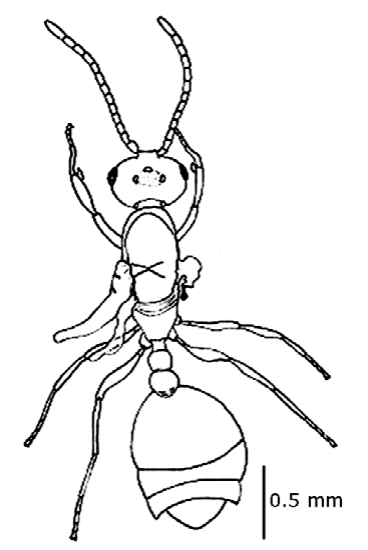
*Monomorium floricola* gynandromorph with predominance of male characteristics, but without male genitalia and vestigial wing on the right side. High quality figures are available online.

**Figure 10.**
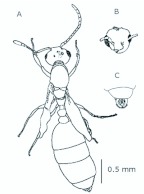
*Monomorium floricola* gynandromorph with ergatogyne characteristics on the left side of the head and elongated gaster. (A) Dorsal view, (B) head frontal view, (C) male genitalia frontal view. High quality figures are available online.

**Figure 11.**
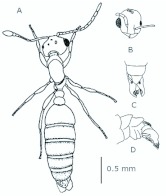
*Monomorium floricola* gynandromorph with ergatogyne characteristics on the left side of the head, alitrunk and gaster. (A) Dorsal view, (B) head frontal view, (C) frontal view of posterior apex of gaster, (D) lateral view of posterior apex of gaster. High quality figures are available online.

**Figure 12.**
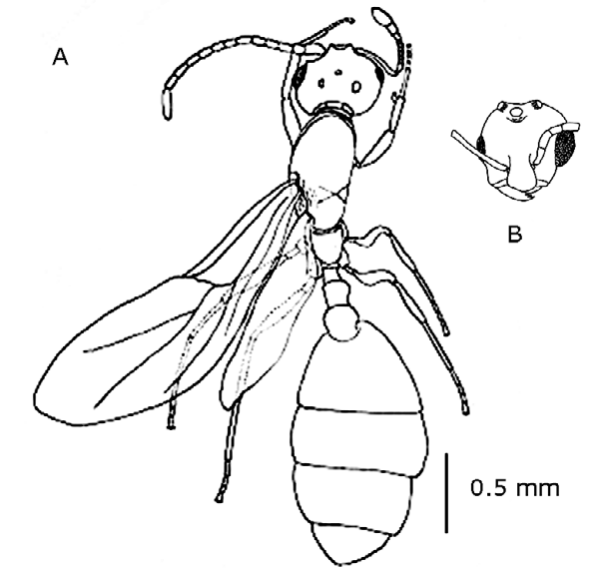
*Monomorium floricola* gynandromorph with ergatogyne characteristics on the right side. (A) Dorsal view, (B) head frontal view. High quality figures are available

**Figure 13.**
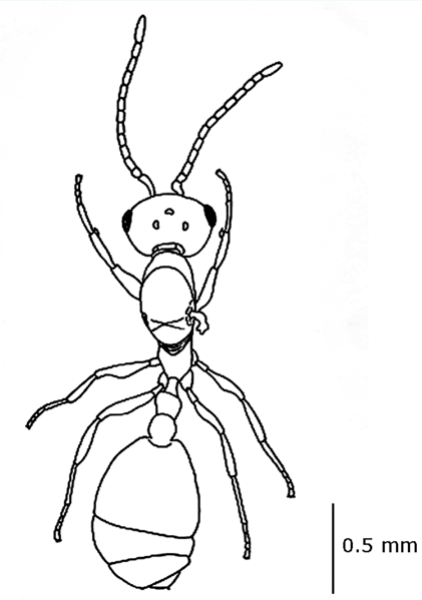
*Monomorium floricola* gynandromorph with male characteristics except by alitrunk and gaster with vestigial wing in one side. High quality figures are available online.

*M. floricola* showed straight bilateral gynandromorphs (Figure 4), but this was only sometimes present in male reproductives. Mosaic combinations were very diverse with either ergatogynes showing male characteristics or males with ergatogynic characteristics. In some gynandromorphs different mandibles sizes were noted (larger on ergatogynes) as well as different eyes, ocelli, antennae, number of segments in the gaster, presence of both male structures, a larger male alitrunk and wings located in either both sides or only one, atrophied or not (Figures 5, 6, 7, 8, 9, 10, 11, 12, 13).

Despite reproductive males having larger thoracic regions, ergatogynes were larger in their overall size. Gynandromorphs showed different sizes in *M. floricola.* They could be as large as ergatogynes, males, mid-sized, or even larger than the reproductives.

**Figure 14.**
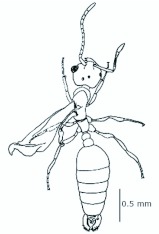
*Monomorium floricola* ergatandromorph. High quality figures are available online.

**Figure 15.**
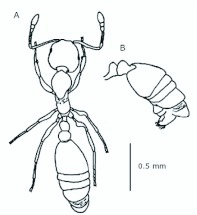
*Monomorium floricola* ergatandromorph with predominance of worker characteristics. (A) Dorsal view, (B) gaster lateral view. High quality figures are available online.

**Figure 16.**
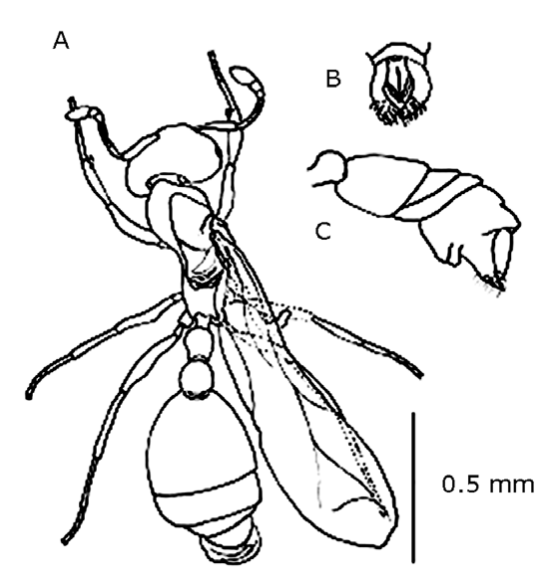
*Monomorium floricola* ergatandromorph with predominance of male characteristics on the alitrunk and gaster with male genitalia. (A) dorsal view, (B) male genitalia frontal view, (C) gaster lateral view. High quality figures are available online.

**Figure 17.**
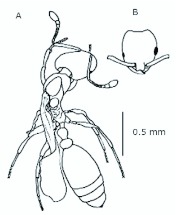
*Monomorium floricola* ergatandromorph with predominance of male characteristics on the alitrunk, with bigger left eye and male genitalia. (A) dorsal view, (B) head frontal view. High quality figures are available online.

**Figure 18.**
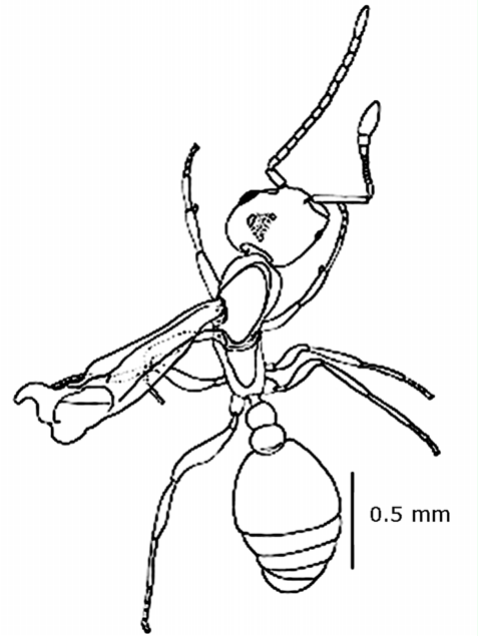
*Monomorium floricola* ergatandromorph with predominance of male characteristics on the left side. High quality figures are available online.

Ergantandromorphs were the size of workers or intermediate sizes between workers and males. They were usually a mosaic with few or many male morphologic characteristics. Most of them have an ergatogyne-like head, which may have a larger male-like-eye. One of them had a male head and the other had only the left side of a male head, but it presented two ocelli on that side (Figures 14–18).

If the categories used by Berndt and Eichler (1987) are adopted, the specimens found in *M. floricola* colonies may be gynandromorphs “stricto sensu”, androginomorph/ ergatandromorph, or ergatandromorph/androergatomorph.

The time for gynandromorphic development from egg to adult was the same relative to reproductive males or ergatogynes, but for ergatandromorphs it was much longer than workers that showed a mean development of 63.5 days.

## Discussion

Similar variety and occurrence of gynandromorphs has been found in few ant species, but in the majority of species only one gynandromorph specimen was described for each species. For example, *L. (Acanthomyops) latipes* (Wheeler 1919), S. *invicta* (Hung et al. 1975), *S. aurea* (Cokendolpher and Francke 1983), *P. dentata* (Jones and Phillips-Jr. 1985), *P. occidentalis* (Taber and Francke 1986), *M. sabuleti* (Scupola 1994), and *Smithistruma* (Munsee 1994). Such differences could be related to the fact that the cited authors observed gynandromoprhs from colonies collected in the field. This is different from the work reported in this study in which colonies were kept under laboratory conditions for 4 years. Kinomura and Yamauchi (1994) found many gynandromorphs that were obtained from the natural population of *Vollenhovia emeryi* (microgyna form) in Gifu, Japan. They were primarily male, most had the thorax and gaster of males and the head contained tissues partially feminized to varying degrees.

For some of the authors cited above, gynandromorphism was caused by shock treatment resulting from stress due to heat. This is unlikely to have occurred in the *M. floricola* colonies in our study because gynandromorphs and ergatandromorphs were observed throughout the year, both in the Entomology Laboratory at Instituto Biológico in São Paulo and in the Center of Studies of Social Insects of UNESP- Rio Claro, where the temperatures did not vary greatly.

It is difficult to determine the cause of gynandromorphism in the observations described here, but trophic disturbance, as considered by Donisthorpe (1929), is unlikely to be the factor that promoted the phenomenon on *M. floricola* because all the nests were fed adequately. As the gynandromorphs were found throughout the year in the artificial nests the authors suggest that gynandromorphism in this species is not a rare phenomenon. Workers have been seen to kill such organisms and in the field the same may occur. Sex is determined in eggs and feeding only influences the caste determination of workers and queens. Further studies are necessary to elucidate the causes of gynandromorphism in this species.
